# Circular from Bureau on Legislation, Etc., American Institute

**Published:** 1883-01

**Authors:** John C. Morgan

**Affiliations:** Chairman; Office of the Chairman of the Committee on Legislation, American Institute of Homœopathy, 1706 Green Street; Philadelphia


### CIRCULAR FROM BUREAU ON LEGISLATION, ETC., AMERICAN INSTITUTE.

OFFICE OF THE
CHAIRMAN OF THE COMMITTEE ON LEGISLATION,
AMERICAN INSTITUTE OF HOMŒOPATHY,
1706 Green Street.

PHILADELPHIA, *September 15th, 1882.*

To M. D. DEAR DOCTOR:—The above-named Committee for the current year of the Institute consists of the following members: John C. Morgan, M. D., Philadelphia, *Chairman;* A. I. Sawyer, M. D., Monroe, Mich.; J. P. Dake, M. D., Nashville, Tenn.; F. H. Orme, M. D., Atlanta, Ga.; E. C. Franklin, M. D., Ann Arbor, Mich.; I. Tisdale Talbot, M. D., Boston, Mass.; J. C. Budlong, M. D., Centredale, R. I.; George F. Roberts, M. D., Chicago, Ill.; Philo G. Valentine, M. D., St. Louis, Mo.; Ambrose S. Everett, M. D., Denver, Col.; C. B. Currier, M. D., San Francisco, Cal.; G. W. Pope, M. D., Washington, D. C. The *Districts* remain as last year, very nearly.

Your attention is respectfully called to the necessity of securing numerous helpers within your district in order to carry out our work, particularly in securing Congressional votes, as hereinafter explained.

The most important duty now before the Committee is that imposed by the vote of the American Institute at its late session, instructing us to press the demand for equal rights in the medical corps of the United States Army and of the Civil Service.

Pursuant thereto, the Chairman prepared a "joint resolution" for presentation to both Houses of Congress. This was amended by the Hon. Charles O'Neill, M. C., of Philadelphia, and presented by him (by unanimous consent) in the House of Representatives, and by the Hon. J. Donald Cameron (also by unanimous consent), in the Senate. In each House it was read twice and referred to the appropriate committee, in whose custody it remains and whose active support must be now secured.

The following is a copy of the "Joint Resolution" in question:

"House Res. 259;" July 17th, 1882.
"Senate " 96;" July 14th, 1882.

"JOINT RESOLUTION—relative to Schools of Medical Practice in the United States and the graduates thereof:

"*Resolved, by the Senate and House of Representatives of the United States of America, in Congress assembled,* That it shall be a misdemeanor, punishable by a fine of five hundred dollars and dismissal from office, for any officer of the United States Government, Civil, Military, or Naval, to make discrimination in favor of or against any school of medical practice, or its legal diplomas, or its duly and legally graduated members, in the examination and appointment of candidates to medical service in any of the departments of the Government.

"SEC. 2. That all such examinations shall be open to the attendance and witness of all physicians, citizens of the United States; and that duly certified copies of the complete records of all the details of said examinations shall be placed on file in the Office of the Librarian of Congress, subject to the inspection and use of members of Congress."

On the introduction of this measure, the journals of the Old School showed much alarm and opposition, even abusing the Honorable Senator for his action. On the other hand, the allopathic liberals have contended for its propriety. We thus learn that we must prepare for a sharp contest.

The first step will be to secure immediate and favorable consideration thereof by each of these Committees; to which end the several gentlemen of the committees named should be duly informed of its nature, and their approval individually sought by the prompt and earnest personal efforts of physicians and others in their respective districts. The resulting recommendations of these Committees will doubtless largely shape the action of the House and Senate. The consequence to Homœopathy will be immense.

The second step will be to obtain (duplicate) signatures in large numbers, everywhere, to the annexed petition to the two Houses. The petitions should be forwarded *as soon as possible*, through the Chairman of this Committee or otherwise, before the *opening* of the winter session, in order that the resolution may obtain an *early* consideration in each House *for third reading and passage.*

The third step will be to secure the influence of each and every member of Congress, through the personal appeals of our friends and theirs, united with those of all the excluded systems of practice in their own districts, letting all know that scientific medicine fears nothing and can lose nothing thereby. Our societies, everywhere, should pass and forward *resolutions sustaining* members who favor our cause.

The fourth step will be to endeavor to secure from the President of the United States a like favorable consideration and *his signature.*

Every person who shall see this circular is particularly requested to take notice of whatever of these steps he or she may be able to further in any way, and to constitute a committee for that purpose. *Let all action be immediate!*

It is to be borne in mind, and duly urged, that the British Medical Act of 1858 (Section xxiii) is of like tenor as respects the impartial licensing of medical men in civil life, going so far as to revoke the licensing power of institutions vested therewith as a penalty for violation of the said prohibition. This act forms an undoubted precedent in *law.* The *equity* is undeniable. (See *British Journal of Homœopathy*, April 1st, 1882.)

JOHN C. MORGAN, M. D., *Chairman.*

The following is the form of heading of the Petition to be circulated and signed, and of which copies will be furnished by the Chairman:

*To the Honorable, the Senate and House of Representatives of the United States of America, in Congress Assembled:*

The undersigned, your petitioners, respectfully represent to your honorable bodies,

1. That the theory and practice of medicine is a matter of great importance to the Army, the Navy, and the Civil Service of the United States;

2. That at the present time the opinions and practice of physicians of equal learning, ability, and honesty differ so widely as to divide them into sects, such as those commonly called allopathic and homœopathic;

3. That one of these sects, calling itself "regular," has now, and has always held, absolute medical control of all Departments of the Government Service, thus compelling all Government employees to submit to its arbitrary choice of medical treatment.

4. That no candidate for appointment to medical service under the Government, who avowed his belief in any other system of medical practice than that called "regular," however learned and well qualified in other respects, has heretofore been accorded an appointment, or even an examination for the same, in any Government service.

5. That such discrimination in favor of one medical system against all the others, equally high in the confidence of the people of the United States, is an evident usurpation of powers not granted to the said public servants by law, and therefore tacitly prohibited to them.

6. That your petitioners, patrons of all systems of medical practice, including the so-called "regular" itself, do earnestly pray that such unjust and injurious discrimination be hereafter prohibited by law of Congress in some form, such as the Joint Resolution now before your honorable bodies, viz.: Senate Resolution (1st Session), No. 96, and House Resolution, No. 259, of July 14th and 17th, last, respectively; and that all *qualified* physicians be thus made *equal before the law* in the Government Service.

And your petitioners will ever pray, etc.

